# Local Duplication of TIR-NBS-LRR Gene Marks Clubroot Resistance in *Brassica napus* cv. Tosca

**DOI:** 10.3389/fpls.2021.639631

**Published:** 2021-04-08

**Authors:** Piotr M. Kopec, Katarzyna Mikolajczyk, Ewa Jajor, Agnieszka Perek, Joanna Nowakowska, Christian Obermeier, Harmeet Singh Chawla, Marek Korbas, Iwona Bartkowiak-Broda, Wojciech M. Karlowski

**Affiliations:** ^1^Department of Computational Biology, Institute of Molecular Biology and Biotechnology, Faculty of Biology, Adam Mickiewicz University Poznan, Poznan, Poland; ^2^Department of Genetics and Breeding of Oilseed Crops, Plant Breeding and Acclimatization Institute-National Research Institute, Poznan, Poland; ^3^Institute of Plant Protection - National Research Institute, Poznan, Poland; ^4^Department of Plant Breeding, Justus-Liebig-Universitaet Giessen, Giessen, Germany; ^5^Helmholtz Zentrum München, German Research Center for Environmental Health, Neuherberg, Germany

**Keywords:** *Brassica napus*, *Plasmodiophora brassicae*, Oxford Nanopore, TNL, RNA-Seq, QTL, resistance, duplication

## Abstract

Clubroot, caused by *Plasmodiophora brassicae* infection, is a disease of growing importance in cruciferous crops, including oilseed rape (*Brassica napus*). The affected plants exhibit prominent galling of the roots that impairs their capacity for water and nutrient uptake, which leads to growth retardation, wilting, premature ripening, or death. Due to the scarcity of effective means of protection against the pathogen, breeding of resistant varieties remains a crucial component of disease control measures. The key aspect of the breeding process is the identification of genetic factors associated with variable response to the pathogen exposure. Although numerous clubroot resistance loci have been described in *Brassica* crops, continuous updates on the sources of resistance are necessary. Many of the resistance genes are pathotype-specific, moreover, resistance breakdowns have been reported. In this study, we characterize the clubroot resistance locus in the winter oilseed rape cultivar “Tosca.” In a series of greenhouse experiments, we evaluate the disease severity of *P. brassicae*-challenged “Tosca”-derived population of doubled haploids, which we genotype with Brassica 60 K array and a selection of SSR/SCAR markers. We then construct a genetic map and narrow down the resistance locus to the 0.4 cM fragment on the A03 chromosome, corresponding to the region previously described as *Crr3*. Using Oxford Nanopore long-read genome resequencing and RNA-seq we review the composition of the locus and describe a duplication of TIR-NBS-LRR gene. Further, we explore the transcriptomic differences of the local genes between the clubroot resistant and susceptible, inoculated and control DH lines. We conclude that the duplicated TNL gene is a promising candidate for the resistance factor. This study provides valuable resources for clubroot resistance breeding programs and lays a foundation for further functional studies on clubroot resistance.

## Introduction

*Plasmodiophora brassicae* Wor., an obligate, soil-borne parasite of crucifers (Brassicaceae), is an agent responsible for clubroot disease. During the two-stage infection (Kageyama and Asano, 2009; Liu et al., 2020), the pathogen hijacks multiple nodes of host metabolism and induces hyperplasia and hypertrophy of the underground organs leading to a prominent galling. The galls act as a major physiological sink that supports the proliferation and development of the pathogen while reducing the fitness of the host (Malinowski et al., 2019). Deformations of the root system impair the plant’s capacity for water and nutrient uptake, leading to growth retardation, wilting, and premature, non-optimal flowering (Korbas et al., 2009). Many important crops cultivated worldwide, including oilseed rape (*Brassica napus*), belong to the Brassicaceae (Dixon, 2007). Clubroot disease has been becoming a global problem of increasing economic impact in cruciferous crops and has been ranked under the top 10 most significant worldwide threats to oilseed rape production (Dixon, 2009; Zheng et al., 2020). An infection of oilseed rape was shown to cause up to 60% loss of yield at relatively low spore densities, and total yield failure at a higher pathogen pressure (Strehlow et al., 2015). Once introduced, *P. brassicae* is hard to eradicate. Resting spores can live in the soil for up to 20 years (Wallenhammar, 1996), and spread easily via, for example, dirt on farm equipment (Cao et al., 2009). Many protective measures against the pathogen, e.g., crop rotation or chemical control, are of limited efficiency (Hwang et al., 2014). Therefore, the breeding of resistant plant varieties remains a crucial component of clubroot control efforts.

The key aspect of the breeding process is the identification of genetic features associated with plant response to pathogen exposure. Numerous clubroot resistance loci were described in *Brassica* crops (Landry et al., 1992; Figdore et al., 1993; Voorrips and Visser, 1993; Grandclément and Thomas, 1996; Voorrips et al., 1997; Matsumoto et al., 1998; Moriguchi et al., 1999; Suwabe et al., 2003, 2006; Hirai et al., 2004; Laurens and Thomas, 2004; Piao et al., 2004; Rocherieux et al., 2004; Nomura et al., 2005; Saito et al., 2006; Sakamoto et al., 2008; Nagaoka et al., 2010; Kato et al., 2012; Chen et al., 2013; Chu et al., 2013, 2014; Hatakeyama et al., 2013, 2017; Pang et al., 2014, 2018; Zhang et al., 2014; Fredua-Agyeman and Rahman, 2016; Lee et al., 2016; Huang et al., 2017; Yu et al., 2017; Dakouri et al., 2018; Hirani et al., 2018; Nguyen et al., 2018; Peng et al., 2018; Laila et al., 2019; Choi et al., 2020; Farid et al., 2020; Karim et al., 2020) and are reviewed in (Neik et al., 2017; Lv et al., 2020).

Several genetic studies were performed in *B. napus.* Manzanares-Dauleux et al. (2000) described a major resistance gene *Pb-Bn1* on the A04 chromosome and two quantitative loci on A04 and C05 chromosomes. The resistance derived from the DH ECD-04 line (selected from *Brassica rapa* subsp. rapifera), which was utilized in many breeding programs for the development of clubroot-resistant cultivars, including winter oilseed rape “Mendel,” was mapped to the *CRa/CRb* region on the A03 chromosome (Diederichsen and Sacristan, 1996; Diederichsen et al., 2006; Fredua-Agyeman and Rahman, 2016; Zhang et al., 2016). Werner et al. (2008) mapped 19 QTLs spread across 8 chromosomes. In addition, a couple of association studies were conducted on the *B. napus/P. brassicae* pathogenic model. Li et al. (2016) identified 9 loci, 7 of which were not described previously. Hejna et al. (2019) identified 2 major and 7 minor loci, with the most prominent peak overlapping the *CRa* region. Fredua-Agyeman et al. (2020) identified three genomic hotspots corresponding to *Crr3/CRk/CRd* and *CRa/CRb/CRb^*Kato*^* regions on A03 and *Crr1* region on A08 in a GWAS study of 124 rutabaga accessions from Nordic countries.

Additionally, two resistance genes were cloned thus far: *CRa* (Ueno et al., 2012) and *Crr1* (Hatakeyama et al., 2013). Both genes belong to the TIR-NBS-LRR (TNL; Toll/interleukin-1 receptor-like – nucleotide-binding site – leucine-rich repeat) protein domain family, reported as a key component of effector-triggered immunity (DeYoung and Innes, 2006; McHale et al., 2006).

Despite a seemingly ample collection of resistance loci, continuous updates on the sources of resistance are necessary. *P. brassicae* shows pathogenic specialization, and the host’s resistance genes often confer immunity to only subsets of pathotypes. Moreover, the breakdown of clubroot resistance in the case of some *P. brassicae* pathotypes has been repeatedly reported (Diederichsen et al., 2014; Strelkov et al., 2016).

In this study, we map the resistance locus of the Swedish resynthesis-derived winter-type oilseed rape cultivar “Tosca” (Happstadius et al., 2003; Diederichsen et al., 2009) to a small region on the A03 chromosome. Using the long-read Oxford Nanopore (ON) sequencing technology, we review the genomic structure of the locus in “Tosca” as well as in susceptible “BRH-1” breeding line. In addition, we perform an RNA-seq experiment to identify infection-induced differentially expressed genes. These data are subsequently linked to the genic composition of the resistance locus. Based on the results, we attribute the “Tosca” resistance phenotype to a locus constitutively expressing a duplicated TNL gene, located within the *Crr3* (Hirai et al., 2004) region, directly upstream of the region homologous to the *CRd* (Pang et al., 2018). This study provides valuable resources for clubroot-resistant rapeseed breeding programs and lays a foundation for further functional studies on clubroot resistance.

## Materials and Methods

### Plant Material

A doubled haploid (DH) segregating population of 250 DH lines was developed by Plant Breeding Strzelce Ltd. (IHAR-PIB Group; division in Borowo) from a cross of a winter oilseed rape (*B. napus*) clubroot resistant cultivar “Tosca” and a susceptible BRH-1 breeding line, using isolated microspore culture technique as described in (Cegielska-Taras et al., 2002; Szała et al., 2020).

### Pathogen Source, Preparation, and Plant Inoculation

Samples of *B. napus* root galls induced by *P. brassicae* were collected from infested oilseed rape fields in Lower Silesian Province in Poland. The inoculum for the greenhouse experiments was prepared by isolating resting spores from the galls. The galls were blended, and the homogenate was filtered through a layer of gauze and centrifuged for 5 min at 3,500 rpm to obtain a clear suspension. Spore density was measured using a 0.1 mm deep, improved Neubauer counting chamber (Marienfeld-Superior) and a bright field microscope (Olympus BX 50). The density was adjusted to 1 × 10^8^ spores/ml. For inoculation, each experimental pot containing five 1-week-old seedlings was watered with the spore suspension. The same batch of inoculum was used in all experiments. Additionally, to assess the *P. brassicae* pathotype, galls from 25 DH lines were collected and individually processed into a set of spore suspensions. *P. brassicae* pathotype of every suspension was classified using the Somé system (Some et al., 1996).

### Experimental Design and Conditions

The greenhouse experiments were performed between April 2018 and August 2019 in the Research Centre of Quarantine, Invasive and Genetically Modified Organisms – Institute of Plant Protection National Research Institute. The experiment followed the principle of augmented design. The plants were grown in a series of 6 temporally successive blocks (batches). Each of the batches included around 60 test DH lines, augmented with 8 reference lines (“checks”) – 6 phenotypically extreme DH genotypes that were selected from the first experiment and parental lines.

For every line, 15 plants were grown in 3 pots: 2 pots for treated (inoculated) and 1 pot for untreated control, 5 plants each. Pots were randomly distributed in 4 trays for treated and 2 trays for untreated control plants. Separate, fixed trays were used for treated and untreated plants to avoid water or soil-borne contamination. The soil pH value was 6.0. The temperature (±0.5°C) was set to 18°C/16°C day/night regime for the first 2 weeks of cultivation, and then elevated to 20°C/18°C. The photoperiod was set to a 14 h/10 h light/darkness scheme. The air humidity (±3%) was 60%. Soil humidity was kept in the range between 60 and 70%.

Despite the controlled experimental conditions, we observed a significant batch effect – seasonal phenotypic variability among the analyzed DH lines. Therefore, an additional experiment was carried out including more lines in one, common batch (242, including the checks) at the expense of the number of tested plants per line (5 instead of 10). Additionally, to promote the infection by *P. brassicae*, the temperature was elevated to 20°C for the first 2 weeks and 24°C/20°C for the next five.

### Phenotyping and Phenotypic Data Analysis

After 7 weeks of growth (42 days after inoculation), the plants were phenotyped for classical underground morphological symptoms of clubroot disease. Each plant was removed from the ground and washed with water. The degree of infection (DOI) was evaluated on a 4-degree scale (Vigier et al., 1989), where 0 indicates a healthy root system, 1 refers to 1–10% of root system altered (small galls on lateral roots), 2 denotes 11–50% root system altered, and 3 describes 51–100% root system altered. The disease index (DI) for each genotype by batch was then calculated by obtaining the arithmetic mean of the DOI and rescaling it to the percent scale.

To obtain the DI over the entire experiment, adjusted for the batch effect (phenotypic variability between the greenhouse runs), the DOI data were fit into a linear mixed model using the lme4 library (Bates et al., 2015) for R (R Core Team, 2020):

(1) $${\text{P}}_{i\,j}=\mu +{\text{g}}_{i}+{\text{B}}_{j}+{\left(\text{gb}\right)}_{i\,j}+{\text{e}}_{i\,j}$$

where P*_*ij*_* stands for the phenotype of the *i*th genotype in the *j*th batch, μ is the general mean of the experiment, g*_*i*_* is the random effect of the *i*th genotype, B*_*j*_* is the fixed effect of the *j*th batch, (gb)*_*ij*_* is the random effect of the interaction between the *i*th genotype and *j*th batch, and e*_*ij*_* is the error term. Next, the conditional mode (Best Linear Unbiased Prediction; BLUP) of the genotype was obtained. The BLUP-DI values were used in a subsequent QTL mapping.

### Heritability Estimation

Broad sense heritability (*H*^2^) of the DOI was estimated after (Stahl et al., 2019) following the concept of (Piepho and Möhring, 2007), with the equation:

(2) $$H^{2}=\frac{\sigma_{G}^{2}}{\sigma_{G}^{2}+S\,E^{2}}$$

where $\sigma_{G}^{2}$ is the genetic variance, derived from a full random model (Eq. 3) and *SE*^2^ is the squared standard error of the difference between the means, derived from a mixed model (Eq. 4). The analysis was conducted using the R packages lmerTest (Kuznetsova et al., 2016), lsmeans (Lenth, 2016), and lme4 (Bates et al., 2015).

(3) $${\text{P}}_{i\,j}=\mu +{\text{g}}_{i}+{\text{b}}_{j}+{\left(\text{gb}\right)}_{i\,j}+{\text{e}}_{i\,j}$$

where P*_*ij*_* stands for the phenotype of the *i*th genotype in the *j*th batch, μ is the general mean of the experiment, g*_*i*_* is the random effect of the *i*th genotype, b*_*j*_* is the random effect of the *j*th batch, (gb)*_*ij*_* is the random effect of the interaction between the *i*th genotype and *j*th batch, and e*_*ij*_* is the error term.

(4) $${\text{P}}_{i\,j}=\mu +{\text{G}}_{i}+{\text{B}}_{j}+{\left(\text{gb}\right)}_{i\,j}+{\text{e}}_{i\,j}$$

where P*_*ij*_* stands for the phenotype of the *i*th genotype in the *j*th batch, μ is the general mean of the experiment, G*_*i*_* is the fixed effect of the *i*th genotype, B*_*j*_* is the fixed effect of the *j*th batch, (gb)*_*ij*_* is the random effect of the interaction between the *i*th genotype and *j*th batch, and e*_*ij*_* is the error term.

To investigate the reliability of each of the batches, their influence on the *H*^2^ was assessed by recalculating the *H*^2^ with a leave-one-out approach.

### Genotyping

The plants were genotyped using The *Brassica* 60 K *Illumina Infinium*^TM^ SNP array (Clarke et al., 2016) and a set of SSR and SCAR markers of known clubroot resistance loci (Supplementary Table 1). For *Brassica* 60 K genotyping, the plant material collected from young leaves was sent to the commercial service provider TraitGenetics in Gatersleben (Germany) for DNA isolation and further processing. For SSR/SCAR analysis, the DNA was extracted from young leaves using a modified CTAB method (Doyle and Doyle, 1990). PCR amplification products were visualized on a 1.5% agarose gel (SCAR) and using the ABI PRISM 3130 OXL capillary electrophoresis (SSR).

### Filtering of Genotyping Data

To check for duplicates, the lines were clustered with complete linkage based on Jaccard’s distance. Lines with <0.05 distance were regarded as duplicates, and only one of them (randomly selected) was used in further analyses. Lines with more than 0.02% of heterozygous calls were discarded. Homomorphic (>95%) markers and markers with distorted segregation patterns (1:3) were also removed from further analyses. Redundant markers were binned.

### Genetic Map Construction and QTL Mapping

A genetic map was constructed using the R/qtl package (Broman et al., 2003). For ordering the markers, the R/TSPmap program was used (Monroe et al., 2017). QTL Mapping was conducted with Haley-Knott regression implemented in the scan1 function of the R/qtl2 (Broman et al., 2019) package. log10(*p*) significance cutoff was determined using a permutation test with *n* = 1,000.

### Genome Sequencing of Parental Lines

Genomic DNA from parental lines was sequenced using ON technology. DNA from young leaves was extracted following a protocol described by Chawla et al. (2020). The libraries were prepared using the SQK-LSK109 kit, following the manufacturer’s recommendations, and sequenced on R9.4.1 Flow Cells.

### Genome Sequencing Data Analysis

The *B. napus* reference genomes used for the study were: Darmor-*bzh* v4.1 (Chalhoub et al., 2014), deposited on EnsemblPlants as AST_PRJEB5043_v1; Express 617 assembly v1 (Lee et al., 2020); reference pan-genome v0 (Song et al., 2020). Darmor-*bzh* genes within the mapped resistance locus were functionally classified using Pannzer2 (Törönen et al., 2018).

Base calling from ON signals was performed using Guppy, and raw reads were mapped to the reference genomes with minimap2 (Li, 2018) with -x map-ont parameters, and filtered for uniquely mapping reads with samtools (Li et al., 2009) using -q 60 option. Local SNV calling was performed using longshot (Edge and Bansal, 2019), with default parameters. SV calling was executed using sniffles (Sedlazeck et al., 2017), with –min_support 5 option. The potential effect of the variants differing parental accessions was determined with the SnpEff (Cingolani et al., 2012).

The reads overlapping the TNL gene on the Express 617 *B. napus* genome assembly were extracted from the raw sequence file, assembled using Redbean (wtdbg2; Ruan and Li, 2020), and polished once using the wtpoa-cns tool.

### Transcriptome Sequencing

For transcriptomic experiments, one resistant and one susceptible DH line were selected. The roots of two biological replicates per line of infected and control plants were harvested on the day of phenotyping (7 weeks after inoculation), immediately frozen in liquid nitrogen, and stored at −80°C. The tissue was blended in liquid nitrogen using a mortar and pestle. The total RNA was extracted with the Qiagen Plant RNeasy kit. TruSeq mRNA strand-specific libraries were prepared and sequenced on Illumina NovaSeq600 in a 2 × 150 bp paired-end layout. Library preparation and sequencing were conducted by Macrogen.

### Reconstruction of the TNL Genes-Encoded Transcripts Using RNA-Seq Reads

RNA-seq reads were pooled by DH line and mapped to the Express 617 genomic sequence assembly supplemented with the fragment containing the duplication as a pseudochromosome using STAR (Dobin et al., 2013) with the following parameters: –outFilterMismatchNoverLmax 0.1 –outFilterMismatchNoverReadLmax 0.1 –alignIntronMax 2000 –alignIntronMin 15 –outSAMprimaryFlag AllBestScore. Reads mapping to the pseudochromosome were then assembled using Trinity (Grabherr et al., 2011) with -genome_guided_bam –genome_guided_max_intron 2000 options. Assembled transcripts were re-mapped to the Express 617 reference sequence supplemented with a pseudochromosome with a minimap2 -x splice for validation. ORFs were predicted and translated using NCBI’s ORFfinder^1^. For sequence comparison, the CDS and protein sequences were aligned with EMBL-EBI’s Clustal Omega and EMBOSS Needle (Madeira et al., 2019). The sequence-based prediction of protein domains was carried out with InterProScan (Jones et al., 2014).

### Analysis of Differential Gene Expression

Raw RNA-seq reads were trimmed using Trimmomatic (Bolger et al., 2014) with default options and mapped to the reference genome using STAR with the following parameters: –outFilterMismatchNoverLmax 0.1 –outFilterMismatchNoverReadLmax 0.1 –alignIntronMax 2000 –alignIntronMin 15. The fragments were counted using the featureCounts (Liao et al., 2014) program with -*s* 2 -*p* -*M* flags. The differential expression analysis was performed using limma (Ritchie et al., 2015; Law et al., 2016)/edgeR (Robinson et al., 2010) R packages, following the procedure described by Law et al. (2016). Raw counts were normalized via TMM, and log-CPM values were used for the DE analysis. The fit was processed with limma’s treat() with lfc = 1 parameter, thus genes with fold-change significantly larger than 2 were deemed as differentially expressed. Gene Ontology enrichment analysis was conducted with g:Profiler (Raudvere et al., 2019). To assess the expression of the “Tosca” TNL copies, the reads were mapped to the reference with the fragment containing the duplication attached as a pseudochromosome, and TPM values of TMM normalized counts were calculated.

### Creation of Figures

All plots were generated with r/ggplot2 (Wickham, 2009). Figures were assembled with Inkscape^2^.

## Results

### Phenotyping of “Tosca” × “BRH-1” DH Population

**FIGURE 1:**
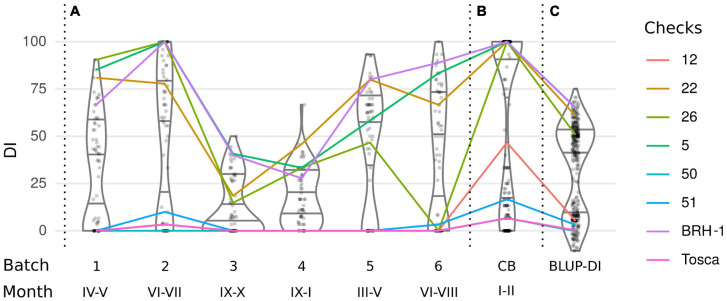
Distribution of the Disease Index (DI) in **(A)** a series of phenotyping experiments, **(B)** joint phenotyping of 240 lines in conditions promoting pathogenesis, and **(C)** a cumulative estimation of the DI-BLUP. The color lines connect DI’s of 8 checks tested in each experiment.

To identify the locus harboring resistance to clubroot disease in the “Tosca” winter oilseed rape cultivar, a mapping population of 250 DH lines was developed in a cross with a susceptible, double-low line BRH-1. For phenotyping experiments, the lines were divided into 7 groups and tested separately in controlled environmental conditions. In addition to the tested DH lines, each experimental batch contained a set of referential “checks” – both parents and 6 DH lines used in all experiments (Figure 1, colored lines). These 6 lines were identified in the first experiment as showing contrasting phenotypes (resistant or susceptible) and no visible developmental abnormalities. In each of the experiments, 1-week-old seedlings were inoculated with a suspension of *P. brassicae* spores prepared from the pathotype-P3-dominant environmental sample. After a total of 7 weeks of growth, the plants were examined for infection-induced morphological pathologies of roots and evaluated on a 4-step severity-dependent scale. For every line, a percent scale DI was calculated.

Analysis of the first 6 batches clearly demonstrated that despite random grouping of the lines the DI distribution was not equal between the batches (Kruskal–Wallis test, *p* < 10–6), with mean DI ranging from 13.13 to 46.86% (Figure 1A and Table 1). Phenotypes of the 8 checks were shifting according to the batches’ trend, showing that the observed differences were independent of the selection of the lines in each of the subsets (Figure 1A). The differences are well explained by seasonal changes, with a higher incidence observed in batches carried out during spring and summer, despite that the plants were grown in controlled greenhouse conditions.

**TABLE 1:** Summary statistics of the DI of the seven batches and BLUP-DI.

**Batch**	**Min**	**Max**	**Mean**	**Median**	**Q_1_**	**Q_3_**	**SD**	**$H_{L\,O\,O}^{2}$**	**Δ*H*^2^**
1	0.00	90.48	30.77	36.67	0.00	55.56	28.87	0.70	0.03
2	0.00	100.00	46.84	57.143	0.00	83.33	40.02	0.65	0.08
3	0.00	50.00	13.11	6.70	0.00	26.70	15.29	0.79	–0.03
4	0.00	62.96	18.89	16.67	5.36	30.56	14.79	0.77	–0.04
5	0.00	92.59	44.23	58.33	0.00	66.67	32.29	0.73	0.00
6	0.00	100.00	38.59	40.00	0.00	73.30	36.02	0.77	–0.02
7	0.00	100.00	63.84	100.00	13.30	100.00	43.97	0.52	0.21
BLUP-DI	–6.45	77.22	37.15	46.87	11.71	56.92	24.32		

*$H_{L\,O\,O}^{2}$ is the *H*^2^ without given batch; $\Delta \,H^{2}=H^{2}-H_{L\,O\,O}^{2}$.*

Since the main goal of this experiment was to identify DH lines with the strongest resistant phenotype, we decided to repeat the tests using growing conditions that promote *P. brassicae* infection. Therefore, in the last 7th batch (Figure 1B), we have included most (240 out of 250) of the DH lines and increased the temperature by 2°C (for details see section “Materials and Methods”). In all 7 batches, the DI values followed a clear bimodal distribution, suggesting that the majority of the phenotypic effect is linked to a single locus. To adjust the DI for the batch effect for QTL mapping, the phenotyping data were fit to a linear mixed model and the BLUP of the genotypic effect (BLUP-DI) was obtained (Figure 1C). The estimated broad-sense heritability of the disease severity for the entire experiment was *H*^2^ = 0.729.

### Identification of the Resistance Locus by Genetic Mapping

The mapping population was genotyped using the *Brassica* 60 k SNP array (Clarke et al., 2016) and a set of SSR and SCAR markers linked to various clubroot resistance *loci*. Markers showing segregation distortion or high heterozygosity were discarded from further analysis. Segregating “Failed” SNP calls were regarded as potential presence-absence variants (Gabur et al., 2018) and kept in the analysis. The constructed genetic map consisted of 1,406 bins of cosegregating markers distributed among 19 linkage groups corresponding to the 19 chromosomes of *B. napus*. The total length of the map was 1866.1 cM with an average spacing of 1.3 cM and a max spacing of 37.2 cM (Supplementary Table 4).

**FIGURE 2:**
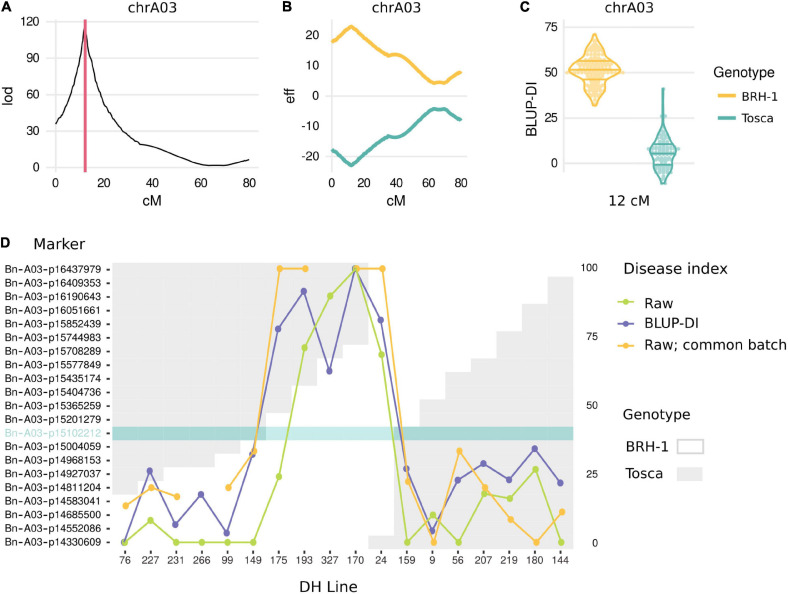
Results of genetic mapping. **(A)** LOD score for QTL presence along the A03 chromosome genetic map. Red lines span Bayes Credible Intervals. **(B)** Effect of “BRH-1” (yellow) and “Tosca” (green) alleles along the A03 chromosome genetic map. **(C)** Phenotype (BLUP-DI) distribution of DH lines carrying “BRH-1” yellow and “Tosca” green alleles at the peak marker. **(D)** Analysis of recombinants. The plot shows a genotype (white: “BRH-1”-inherited, gray: “Tosca”-inherited) at markers surrounding the mapped locus. The phenotype for 18 DH lines recombining in the proximity of the locus is shown as a DI from one of the 1–6 batches, and, if available, 7th, common batch, as well as BLUP-DI. A marker solely explaining the phenotype is highlighted in teal.

QTL mapping on BLUP-DI data revealed a single locus on the A03 chromosome (Supplementary Table 5). Bayes Credible Interval (BCI) for the QTL spanned 0.4 cM between 11.980 and 12.378 cM on the genetic map (Figure 2A and Supplementary Table 5). The same region, although with a larger BCI span, was detected in individual QTL mappings for every phenotyping batch (Supplementary Table 6). No evidence suggested the involvement of other loci affecting the trait. The locus exhibited a large effect, with a 45.65 difference between mean values of BLUP-DI for the bin of markers exhibiting the strongest correlation with the phenotype (Figures 2B,C and Supplementary Table 5). Recombination events in the proximity of the QTL were identified and compared with the phenotypes. This analysis revealed that the state of a single bin of markers, cosegregating with the representative Bn-A03-p15102212 marker at 11.980 cM was sufficient to explain the phenotype (Figure 2D).

To physically anchor the resistance locus, either probe or primer sequences (depending on the marker type) from within the bin with the strongest correlation with the trait were aligned to the Darmor-*bzh* 4.1 reference genome. The closest markers flanking the bin spanned a region of 91,088 bp on supercontig LK031800. However, three of the markers cosegregating with the peak marker mapped to a supercontig LK033659, suggesting that the reference genome has been misassembled.

**FIGURE 3:**
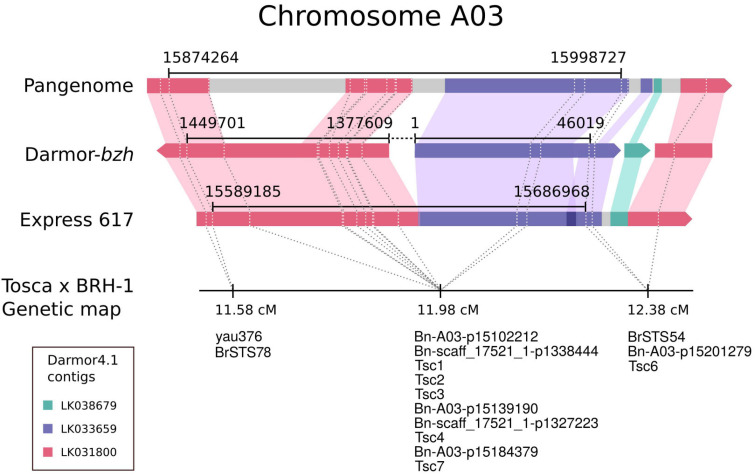
Physical localization of the resistance locus (black line) in different assemblies of the *Brassica napus* genome defined by the bin of cosegregating markers at 11.98 cM. Colored bars represent Darmor-*bzh* 4.1 contigs. Gray color depicts sequences in the pangenome and Express 617 not covered by the Darmor-*bzh* 4.1 contigs.

To resolve this discrepancy, we have mapped both contigs (LK031800 and LK033659) to the recently published *B. napus* reference pangenome (Song et al., 2020) and Express 617 assemblies (Lee et al., 2020). As shown in Figure 3, both contigs map to the same region in the newer long-read-based genomic sequences. The LK031800 (2,713,116 bp) contig maps to two distinct distant parts of the reference sequence that are separated by region corresponding to LK033659 (51,625 bp) and a small contig LK038676 of 4,535 bp in size. For further verification of the Darmor-*bzh* being misassembled, a new set of 6 SCAR markers was designed upstream, within, and downstream of the LK033659 (Tsc, Supplementary Table 2). The PCR results confirmed the observed segregation pattern and were in perfect agreement with the primers’ physical location. Summarizing, the locus defined by the bin of markers (with the representative marker Bn-A03-p15102212) is covered by both new reference *B. napus* assemblies as well as two contigs from Darmor-*bzh* 4.1, but in the latter case, one of the contigs (LK031800) is misassembled (Figure 3).

**FIGURE 4:**
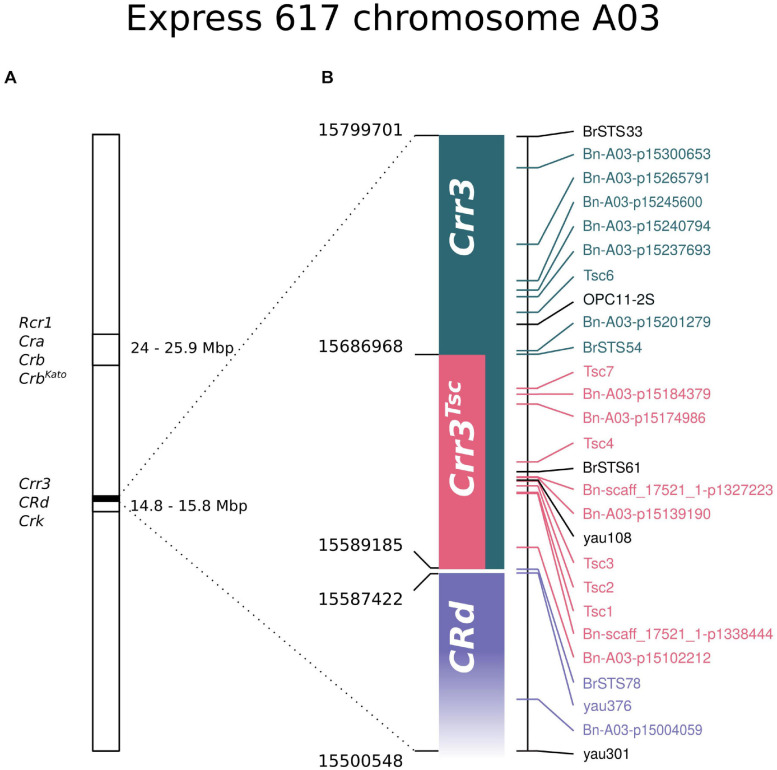
Schematic location of the *Crr3*^*Tsc*^ region in the context of other resistance loci and markers located on Express 617 chromosome A03. **(A)** A general overview of the location of *Rcr1/Cra/Crb/Crb^*Kato*^* and *Crr3/CRd/Crk* regions on the A03 chromosome. **(B)** Zoomed region from the *Crr3/CRd/Crk* fragment (marked with a black box on **A**). Markers labeled with the same color cosegregate in the mapping DH population. No genetic data were obtained for markers indicated with black. The precise start of the *CRd* locus could not be physically mapped onto Express 617 assembly.

Concluding, the genetic factor of resistance to *P. brassicae* infection is located in the region covering 124,463 bp on the reference pangenome, 97,783 bp on the Express 617 assembly, and 118,111 bp on the Darmor-*bzh* 4.1 assembly. Genetic and physical evidence suggests that the mapped resistance locus falls within the region homologous to the *Crr3* locus (Hirai et al., 2004), directly upstream of the region homologous to the *CRd* (Pang et al., 2018; Figure 4). Accordingly, the resistance locus identified in this study is hereafter referred to as *Crr3*^*Tsc*^. The *Crr3*^*Tsc*^ contains 25 annotated protein-coding genes. The full list with functional descriptions is included in Supplementary Table 7.

### Structural Variation Within the Resistance Locus Between “Tosca” and “BRH-1”

**FIGURE 5:**
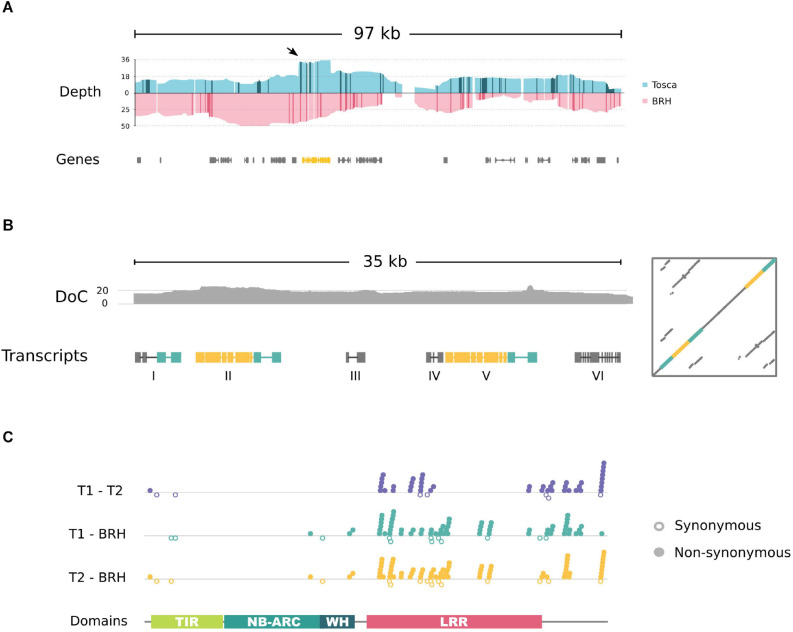
**(A)** Simplified plot showing resistance locus coverage of ONT long reads aligned to the Express 617 reference genome. A sharp, flat peak (indicated by an arrow) in “Tosca” genomic data marks the location of the fragment containing the TNL gene (yellow). Darker colors on the coverage plot represent insertions larger than 10 bp. **(B)** Schematic overview of (i) depth of coverage of ON reads (DoC) and (ii) within the duplicated “Tosca” region. Yellow – a fragment corresponding to the BnaA03g29300D gene; green – homologous fragments of STP6 gene. **(C)** Pairwise alignment-based comparison of CDS sequence variability between TNL homologs in “Tosca” (T1,T2) and “BRH-1.” Synonymous substitutions are marked as empty circles below the axis, non-synonymous are indicated by filled circles above.

To explore in detail the properties of the region covering resistance in the “Tosca” genetic background, we have sequenced the genomes of the parental lines with ON technology. The reads mapped to the Express 617 reference genomic sequence consistently overlapped the resistance locus genomic region (97,783 bp) with an average read coverage of 16.24 for “Tosca” and 28.50 for “BRH-1,” with 93.6 and 91.9% of positions covered with at least 5 reads, respectively (Figure 5A and Supplementary Figure 1).

The long-read mapping results showed differences between the “Tosca” and reference genome assembly. Interestingly, the read coverage depth of the fragment overlapping a TNL gene (BnaA03g29300D) was approximately doubled in comparison to the surrounding sequences in “Tosca,” but not in “BRH-1” (Figure 5A and Supplementary Figure 1). Moreover, neither of the reads spanned the entire gene and both of its flanking regions, and many of them mapped twice to the gene. To further investigate these observations, we have *de novo* reassembled this fragment using exclusively long reads mapping to the gene. The new assembly, supported by 20× average coverage and multiple span-through reads, revealed a 7 kb duplication in the “Tosca”, but not “BRH-1” genome (including a full copy of the TNL gene – described below; yellow box in Figure 5B and Supplementary Figure 2).

**FIGURE 6:**
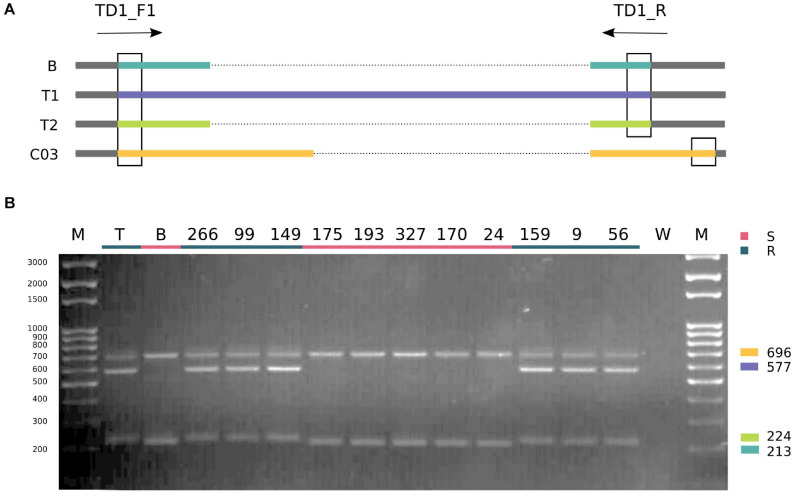
Allele-discriminating PCR. The gel electropherogram **(B)** shows a result of PCR reaction designed as depicted on the schematic **(A)**. B, T1, and T2 stand for BnaA03g29300D copies from “BRH-1” and “Tosca,” respectively. C03 represents a homoeologous region on the C03 chromosome, identical between the parental lines. Expected products are color-coded according to the schematic, and juxtaposed with the gel. Gel lanes: M – size marker, T – “Tosca,” B – “BRH-1,” W – water-containing control reaction, 266-56 – selected lines, recombining in the proximity of the resistance locus. Susceptible lines are underlined with red, resistant with teal. Both “Tosca” alleles (fragments 224 bp and 577 bp) segregated with the resistance.

The duplication identified in “Tosca” was further confirmed using a pair of primers flanking the polymorphic site (TD1_F1/TD1_R, Supplementary Table 2) that generate different product lengths for “BRH-1,” both duplicated “Tosca” TNL paralogs and their homeolog from the C genome in “Tosca” and “BRH-1” (C genome homeologs are nearly identical in both lines; Supplementary Figure 3). PCR reactions performed on the parents and 11 DH lines with varying degrees of infection resistance revealed the expected pattern of bands, with both “Tosca”-specific alleles segregating with the resistance phenotype (Figure 6).

The duplicated region covers the entire TNL gene and a fragment homologous to STP6 Figure 5B and Supplementary Figure 2. In Darmor-*bzh*, the STP6 fragment is annotated as a distinct gene (BnaA03g29290D), while in Express 617 assembly, the TNL and STP genes are merged into a single entity (A03p030030.1_BnaEXP). Neither the TNL nor the STP fragment is annotated in the scaffoldA03 of the pangenome.

Our RNA-seq data analysis suggests that in both copies the TNL and STP fragment form a single transcription unit; however, the CDS terminates before reaching the STP fragment. The duplicates are separated by a 12 kbp spacer, containing two ∼1.2 kbp transcribed, spliced regions. Blast search of genomic and transcriptomic sequences of both transcribed fragments did not provide any conclusive results. Transcript variants of the transcribed regions contained fragmented ORFs (up to 117 aa in length) with limited similarity to known proteins.

Additionally, the duplicated genes (including the STP fragment) differ in their splicing structure, producing transcripts with 8 and 9 exons, respectively. They share more than 90% identity on the genomic sequence level, with most differences situated downstream of the TNL encoding ORF. On the transcript level, the similarity is 90.9% (4529/4982). The protein sequences are identical in 94.9% (1051/1108) and similar in 96.3 (1067/1108). The alignment has 13 gaps (1.2%), with 10 located at the very end.

### Sequence Variation Within Resistance Locus

The location and molecular effect of variants differing between “Tosca” and “BRH-1” were assessed using SNPeff with reference to the Express 617 assembly. The region covering resistance to *P. brassicae* infection contains 1521 polymorphic sites: 1327 SNPs, 61 indels, 1 small duplication, and 132 mixed-type variants. The large, duplicated region, covering the TNL gene found in the “Tosca” genome, was omitted from the SNPeff analysis and evaluated separately.

Most of the detected variants are located within intergenic regions. Exonic and intronic polymorphisms account for 6.15 and 7% of the total sequence variability, respectively. The non-synonymous/synonymous substitution ratio is 0.51. The molecular effect, as defined by SNPeff, was high for 0.25%, moderate for 2.07%, and low for 4.62% of the variants. The remaining differences were classified as modifiers. Both genotypes showed the presence of insertions and deletions, located mainly in introns and intergenic regions. Sequences of insertions larger than 500 bp did not show similarity to any annotated genes. A more detailed, gene-oriented analysis of the SNP effect revealed that among protein-coding genes with detectable expression in at least one of the studied lines, only two genes, namely A03p030010.1_BnaEXP (Darmor-*bzh* BnaA03g29270D) and A03p030120.1_BnaEXP/A03p030120.2_ BnaEXP (Darmor-*bzh* BnaA03g57410D), contain high- effect variants. A03p030120.1_BnaEXP/A03p030120.2_BnaEXP encodes a metallochaperone and carries a splice donor variant in the “Tosca” cultivar. Besides, this gene harbors the highest variability, with 29 missense and 18 synonymous differences between the lines. A03p030010.1_BnaEXP encodes a chaperonin and contains a premature stop codon in the “BRH-1” (Supplementary Table 8).

To reduce the effect of potential ambiguous mapping, the coding sequences of the duplicated “Tosca” TNL genes were compared based on a transcript assembly. The predicted CDS contained a large number of variants between the “BRH-1” gene and both copies from “Tosca” (Figure 5C). Interestingly, as noted before, the “Tosca” paralogs differ considerably at the sequence and gene structure levels (Figure 5C, T1-T2; Supplementary Figure 2).

The *C*-terminal coding fragments have different lengths (60 bp and 30 bp). The lack of 30 bp in the T1 gene results in a frameshift(s) and, consequently, in a different amino acid sequence at the *C*-end of the encoded protein. Apart from the *C*-terminal variance, the sequences differ with regard to 6 synonymous, 36 non-synonymous substitutions, and 9 in-frame deletions (6 and 3 bp long). 35 of the missense variants cluster within and in close vicinity to the LRR domain coding sequence, with the rest of the protein sequence differing only at one position near the *N*-terminus (Figure 5C).

The “BRH-1” TNL homologous gene has the same *C*-terminal composition as the “Tosca” paralog T2 (Figure 5C, T2-BRH). The BRH and T2 genes differ with regard to 1 in-frame deletion (3 bp), 12 synonymous, and 63 non-synonymous substitutions. On the other hand, the BRH and T1 genes, besides the *C*-terminus variance, differ with regard to 15 synonymous and 59 non-synonymous substitutions (Figure 5C, T1-BRH). Similar to the T1–T2 comparison, nearly all differences between “BRH-1” and “Tosca” gene copies are localized near the *C*-terminus and in the LRR domain coding sequence. The cDNA, CDS, and protein sequences of the “Tosca” and “BRH-1” genes are available in the Supplementary Material.

### Differential Gene Expression Analysis of Resistant and Susceptible DH Lines

To further characterize the 25 genes located within the locus harboring resistance to clubroot disease, we have examined the differences in transcript levels between inoculated and non-inoculated control roots from resistant and susceptible DH lines in the context of the global pattern of differentially expressed genes.

RNA-seq comparison of transcript accumulation between non-inoculated control resistant versus susceptible plants showed a differential signal for 1,247 genes, with only one located within the resistance locus – the BnaA03g29270 gene (Supplementary Tables 9,10). This gene shows a significantly higher (logFC = 1.9, adjusted *p*-value ≤ 0.0017) expression level in the resistant line. The gene encodes for a homolog of an *Arabidopsis thaliana* chaperone protein (CCT3).

Subsequently, we have explored differentially expressed genes in roots 46 days after inoculation (DAI) with *P. brassicae*, using a resistant and susceptible line from the mapping population.

In the case of the resistant line, we have identified 111 genes that showed differential transcript accumulation after inoculation (91 up- and 20 down-regulated genes; Supplementary Tables 9, 10). 53 of these genes were differentially expressed only in the resistant line. Most of them fall into three general Gene Ontology classes: chitin metabolism, regulation of growth, and defense response (Supplementary Table 11). None of the differentially expressed genes identified in the resistant line was located within the resistance locus. The nearest gene showing a differential expression pattern – BnaA03g28780D (encoding Hevein-like preprotein, reported to be involved in the defense response against fungi and bacteria) – is located 200 kbp upstream from the locus.

Analysis of the inoculated susceptible line revealed a much high number (6821) of differentially expressed genes (Supplementary Tables 9, 10). Among them, 2,778 were up- and 4,043 were down-regulated. The Gene Ontology-based assignment showed a much more diverse spectrum of molecular functions, among others: oxidative stress response, carbohydrate metabolism, lignin metabolism, chitin metabolism, defense response, auxin signaling (Supplementary Table 12). The KEGG pathway enrichment analysis yielded significant hits for phenylpropanoid biosynthesis, biosynthesis of secondary metabolites, glutathione metabolism, stilbenoid, diarylheptanoid and gingerol biosynthesis, and flavonoid biosynthesis.

Four of the differentially expressed genes were located within the resistance locus: down-regulated germin-like protein (BnaA03g29240D – ortholog of AtGLP8, AT3G05930), up-regulated Sugar Transporter Protein (BnaA03g29310D – ortholog of AtSTP6, AT3G05960), down-regulated Fantastic Four protein (BnaA03g57340D – ortholog of AtFAF4, AT3G06020), up-regulated protein trichome birefringence-like (BnaA03g57390D – ortholog of AtTBL10, AT3G06080). None of them, however, were differentially expressed in the resistant line. Moreover, their expression levels were similar in resistant and susceptible control, non-inoculated plants.

The transcript levels of the TNL gene BnaA03g29300D remained unchanged for “BRH-1” and both “Tosca” copies in both lines after inoculation; however, the “BRH-1” and “Tosca” copies were expressed at relatively high levels in the control and the inoculated plants. The transcript levels of the two “Tosca” copies detected in the resistant line added up to twice the amount of the transcript level of one copy expressed in the susceptible line (Supplementary Table 13).

## Discussion

Based on genetic mapping of a population of 250 DH plants, we were able to identify a single locus conferring resistance to clubroot disease in the winter oilseed rape cultivar “Tosca.” The “Tosca” resistance has a different background than the widely utilized “ECD-04,” introgressed into the “Mendel” cultivar (Diederichsen and Sacristan, 1996; Diederichsen et al., 2006; Fredua-Agyeman and Rahman, 2016), which makes the source relevant in the *B. napus* breeding efforts.

The identified “Tosca” resistance locus, designated as *Crr3*^*Tsc*^, in *B. napus* is located on the A03 chromosome within a previously described *Crr3* locus described in *B. rapa* (Hirai et al., 2004; Saito et al., 2006), which together with *CRk* (Sakamoto et al., 2008; Matsumoto et al., 2012), and *CRd* (Pang et al., 2018) forms a larger cluster of clubroot resistance genetic factors. This cluster has been recently spotted in a GWA study in a panel of *B. napus* ssp. *napobrassica* (rutabaga), which, like “Tosca”, are of Nordic origin (Fredua-Agyeman et al., 2020). The region of ∼750 kbp identified in this GWA study was associated with resistance to *P. brassicae* pathotypes 2B and 8P (classified according to Canadian Clubroot Differential Set), which are subsets of Some’s P2 pathotype (Strelkov et al., 2018). Here, we show that a region of ∼120 kbp of the *Crr3*^*Tsc*^ locus explains the resistance to field isolates consisting of a mixture of pathotypes with the highest prevalence of P3.

The locus was anchored based on a single bin of 11 marker sequences to a region of 97,783 bp of the *B. napus* Express 617 genome assembly. Within the locus, we have identified 25 protein-coding genes. 13 of them were found to be constitutively expressed at the late stage of infection and 4 were found to be differentially expressed between contrasting susceptible and resistant lines of the mapping population. Some of these genes show a functional annotation that makes them interesting candidates to be involved in various stages of *P. brassicae* infection.

Resistance might be expressed constitutively or induced after the initial infection with the pathogen. The non-inoculated control plants showed significant differences in constitutive gene expression patterns, but only 1 out of 1247 was located within the mapped resistance locus. The differentially expressed gene BnaA03g29270D, a homolog of *Arabidopsis thaliana* chaperone protein CCT3, does not offer a direct and evident connection to the mechanism of plant resistance and is unlikely to be involved in resistance expression. The analysis of gene expression affected by the interaction with the pathogen at the later stage of infection provided more interesting candidates. For example, BnaA03g29310D gene which is a homolog of AtSTP6. STPs are monosaccharide/H + symporters that mediate the transport of monosaccharides from the apoplast into the cells (Büttner, 2010). We have observed a significant upregulation of STP6 in infected roots of susceptible, but not of resistant plants. STP family genes, namely STP8 and STP13, have previously been reported to be up-regulated upon clubroot infection in *A. thaliana* (Walerowski et al., 2018), while STP4, STP12, STP1 showed a differential expression pattern in an infected, clubroot-susceptible *Brassica oleracea* cultivar CS-JF1 (Zhang et al., 2019). However, as the expression was affected only in the susceptible line, this gene might have been up-regulated in response to a successful transformation of plant metabolism by the clubroot pathogen during the invasion of the roots. Thus, a potential resistance effect would have to be driven by the inhibition of the expression induction. A similar phenomenon might be responsible for the expression of the gene BnaA03g57390D, harbored within the resistance locus, encoding a homolog of Trichome Birefringence Like 10 (TBL10) protein. This gene was up-regulated in the roots of susceptible, infected plants, but remained constant in the resistant line. TBL proteins are modifiers of the cell wall (Bischoff et al., 2010; Yuan et al., 2016; Gao et al., 2017) and AtTBL10 was found to be involved in O-acetylation of pectin (Stranne et al., 2018). In many reports, pectin hypoacetylation has been linked to increased disease resistance (reviewed in Pauly and Ramírez, 2018), which may explain the lack of TBL10 induction in clubroot defense reaction. Another down-regulated gene from the resistance locus, BnaA03g57340D, is a member of the FANTASTIC FOUR (FAF) protein family. Its expression was down-regulated in susceptible, but not in resistant plants. Overexpression of FAF members was shown to inhibit root growth, which could be rescued by exogenous sucrose (Wahl et al., 2010). Thus, FAF might perform a role in integrating auxin and sugar signaling during infection progression, allowing the pathogen to manipulate the physiological processes of susceptible plants for more efficient infection progression. Another differentially expressed gene from the resistance locus which has been described to be involved in disease resistance expression is BnaA03g29240D – a homolog of Germin-like protein 8 (GLP8). GLPs are well established as an important component of the biotic stress response (Dunwell et al., 2008; Ilyas et al., 2016). In *B. napus*, GLPs are involved in oxidative burst initiation during *Sclerotinia sclerotiorum* infection (Rietz et al., 2012). GLP5 was also found to have higher expression in a line of *B. rapa* carrying the *Rcr1* clubroot resistance gene (Song et al., 2016). In our study, GLP8 expression was highly reduced in the roots of susceptible plants, though it remained constant in the resistant plants.

Because the expression analysis did not reveal a clear, dominant candidate gene located in the resistance locus that could be responsible for the resistance in “Tosca” we have further explored the properties of the mapped genomic fragment by sequencing the parental cultivars with Oxford Nanopore technology. Detailed analysis of the sequencing data showed a high level of polymorphism on a single nucleotide as well as a larger scale. Despite a large number of differences between the parental lines, most of them were located within the non-coding (genic and non-genic) regions. Additionally, the biological impact of the majority of the polymorphisms was predicted to be low in most of the 25 genes. However, the mapping of the ON reads revealed one striking difference – a large duplication in “Tosca” that covered a full copy of the BnaA03g29300D gene. The duplicated gene contains TIR, NB-ARC, and LRR domains, thus belonging to the TNL subclass of NLR genes. Many of these genes are known to be involved directly or indirectly in the recognition of pathogen effector molecules and initiation of downstream defense responses (reviewed in: Dubey and Singh, 2018; de Araújo et al., 2019). As these genes are known to be involved in the very early stages of signaling cascades, they potentially could be differentially expressed in the early stages of the infection process. The identification of a recent copy of the TNL genes to some extent conforms with this presumption. Assuming that recently duplicated genes retain their original function, we may speculate that the effect of enhanced resistance to pathogen infection in “Tosca” is linked with cumulatively elevated expression (2 times) of two copies of the TNL genes. We cannot, however, exclude an alternative possibility that the new copy of the gene acquired new specificity toward the particular *P. brassicae* pathotypes or that both copies are involved in a more complex resistance initiation (see later). So far, two clubroot resistance genes have been cloned, *Crr1a* (Hatakeyama et al., 2013) and *Cra/CRb* (Ueno et al., 2012; Hatakeyama et al., 2017), both encoding TNL proteins.

The identification of the genomic fragment corresponding to the region defined by the peak marker for the resistance locus was not straightforward using the Darmor-*bzh* 4.1 genome assembly. The coverage of the region was not complete and one of the three contigs mapping to this fragment was misassembled. The locus, however, was correctly placed on the long-read-based reference pangenome and Express 617 assemblies. The Darmor-*bzh* 4.1 reference assembly was constructed prior to the advance of long-read sequencing technologies mainly based on Illumina short-read sequencing and is thus highly fragmented (Lee et al., 2020). We have to note that between the submission and publication of this article, an upgraded, Oxford Nanopore-based version of Darmor-bzh genome (v10) was published, in which the region in question is assembled in agreement with the results of our study and other long-read assemblies (Rousseau-Gueutin et al., 2020). Moreover, it has been shown that in *B. napus* more than 50% of known RGA copies do not occur in a single reference genotype assembly but require analysis of pangenome assemblies for detection (Dolatabadian et al., 2020). Thus, our analysis represents a typical example of how the use of long-read sequencing technology and pangenome sequence assemblies allows more efficient dissection of plant disease resistance loci.

Frequent duplications and clusterization of resistance-related NLR genes are a well-established phenomenon (reviewed in de Araújo et al., 2019; van Wersch and Li, 2019). BnaA03g29300D is flanked by homologous STP6 sequences. This configuration may have served as a foundation for homology-dependent duplication events, for example, by unequal crossing-over. Remarkably, both TNL gene copies seem to be functional, i.e., neither underwent pseudogenization. Both are constitutively expressed in 7-week-old plants and the transcripts contain full-length ORF’s. Importantly, the copies harbor a large proportion of polymorphic, non-synonymous sites observed between “Tosca” and “BRH-1,” nearly all of which lay within the pattern-recognizing LRR domain, implicating a strong positive selection acting on this domain. Positive selection promoting rapid sequence changes in the NLR genes, especially within LRR domains has already been frequently reported (Bergelson et al., 2001; Mondragón-Palomino et al., 2002; Yang et al., 2013; Karasov et al., 2014; Gao et al., 2018; Han, 2019). LRR domains are reported to be the major factors determining recognition specificity (reviewed in Padmanabhan et al., 2009); therefore, the apparent differences in the amino acid sequence domain may be responsible for the capability of the “Tosca” to sense the *P. brassicae* elicitors and induce the downstream defense response. Remarkably, the two tandem paralogs in the “Tosca” genome themselves differ significantly in the amino acid sequence of their LRR domains. The differences may allow for a broader range of elicitor recognition or the coordination of a more complex response to infection. The TNL proteins are known to engage in functional homo- and heterodimerization (Williams et al., 2014) and various modes of entanglement in the defense response initiation, which often involves clustered genes (reviewed in de Araújo et al., 2019; van Wersch and Li, 2019). Nonetheless, in the case of previously described clubroot resistance locus *Cra*/*Crb*/*CRb^*kato*^*, which consists of at least six tandemly repeated NLR genes, a majority of the resistance effect is attributed to a single gene, with residual, unexplained effect (Hatakeyama et al., 2017).

Furthermore, the BnaA03g29300D gene is a homolog of *B. rapa* Bra001175, which has been shown to have a higher level of expression in the clubroot-resistant, compared to the susceptible genotype of the *CRd*-carrying line during the early stages of the infection process, at day 13 after inoculation (Pang et al., 2018). In our study, using RNA-seq data, when each of the duplicated genes was tested separately, we could not find statistical differences in BnaA03g29300D expression between resistant and non-resistant inoculated lines and no evidence of induction after inoculation. However, both duplicated genes are expressed at a similar, relatively high level in the roots of “Tosca”-background plants, showing a cumulative 2-times higher transcript accumulation before infection compared to the “BRH-1”-derived, single copy line.

In summary, the search for the genetic background of resistance to *P. brassicae* infection in *B. napus* cv. Tosca revealed a complex picture of genomic and transcriptomic changes. Based on genetic mapping, structural genomics, expression analyses, and functional annotation, we conclude that the TNL gene (BnaA03g29300D) duplication is most likely to be involved in the resistance. Certainly, further experimental tests, including a gene knockout and functional complementation must be conducted to confirm the role of this gene, and/or its duplication, in the resistance against *P. brassicae*.

## Data Availability Statement

The datasets presented in this study can be found in online repositories. The names of the repository/repositories and accession number(s) can be found below: NCBI SRA, BioProject number: PRJNA685314.

## Author Contributions

KM, CO, and WK conceived the research. EJ and AP performed and MK supervised the phenotyping experiments. KM and JN performed and IB-B supervised the SSR and SCAR genotyping experiments and production of the DH line seeds. PK collected, processed, and analyzed the phenotyping, genotyping, and sequencing data and performed association analyses. HSC generated a part of Oxford Nanopore sequencing data. WK supervised and coordinated the whole project. PK and WK wrote the manuscript. All authors contributed to the article and approved the submitted version.

## Conflict of Interest

The authors declare that the research was conducted in the absence of any commercial or financial relationships that could be construed as a potential conflict of interest.

## Footnotes

1 https://www.ncbi.nlm.nih.gov/orffinder/

2 Harrington et al. http://www.inkscape.org/
