# Supplementary figures and images for: Local Duplication of TIR-NBS-LRR Gene Marks Clubroot Resistance in Brassica napus cv. Tosca
Source: Front Plant Sci. 2021 Apr 8;12:639631. doi: 10.3389/fpls.2021.639631 (PMC8082685; doi:10.3389/fpls.2021.639631)

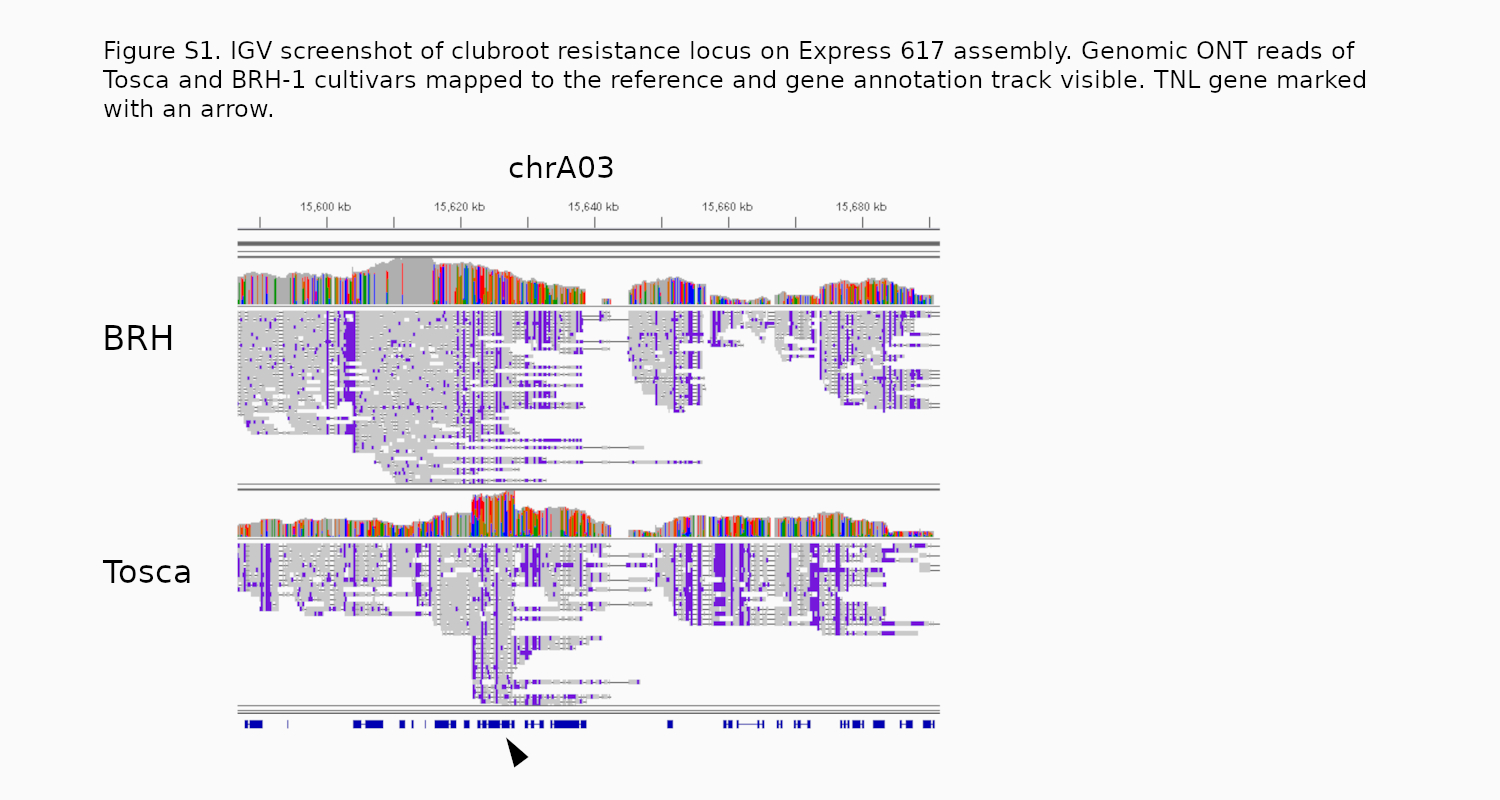

Supplement: Supplementary file 4 [file Image_1.JPEG]

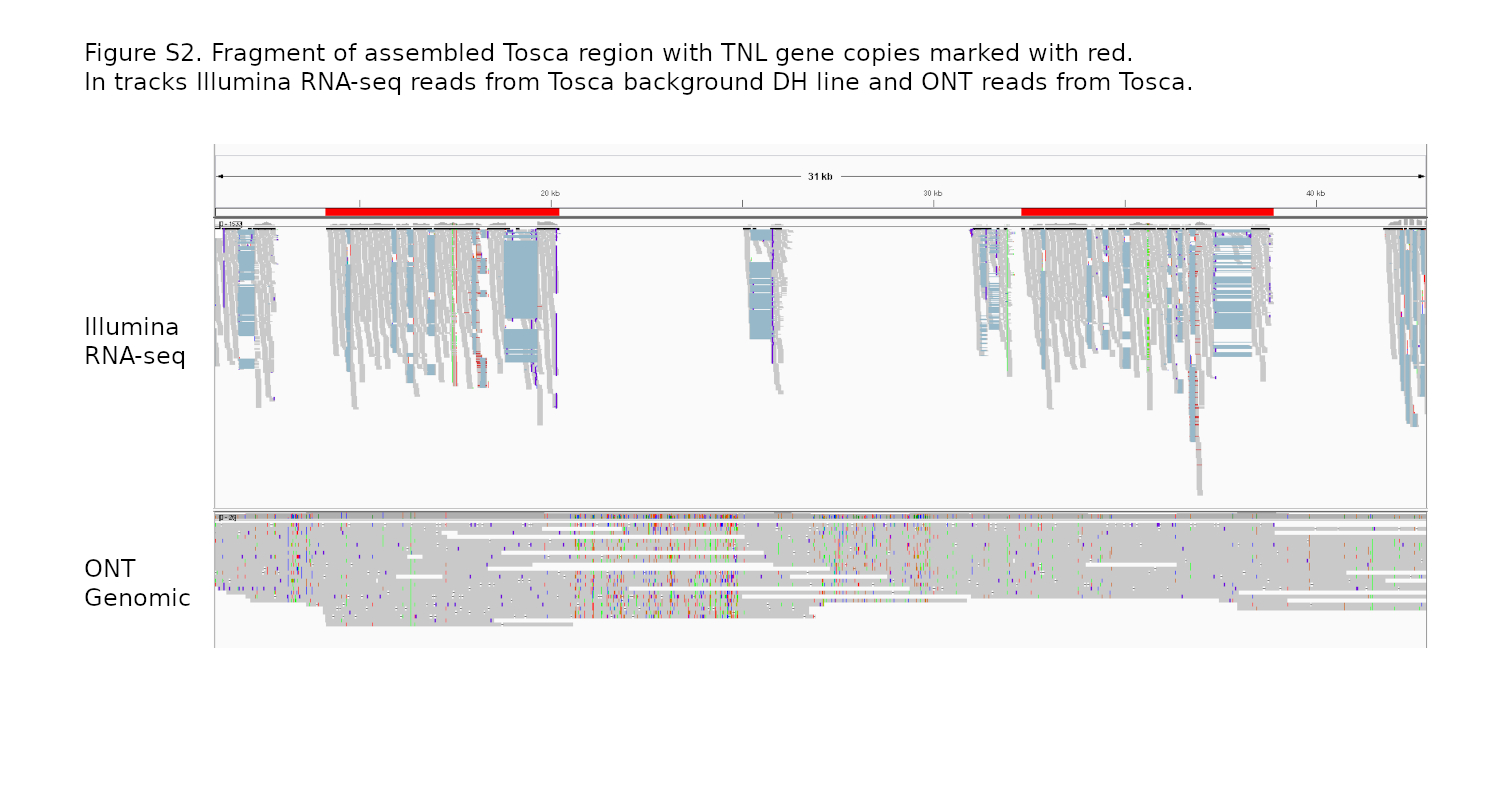

Supplement: Supplementary file 5 [file Image_2.JPEG]

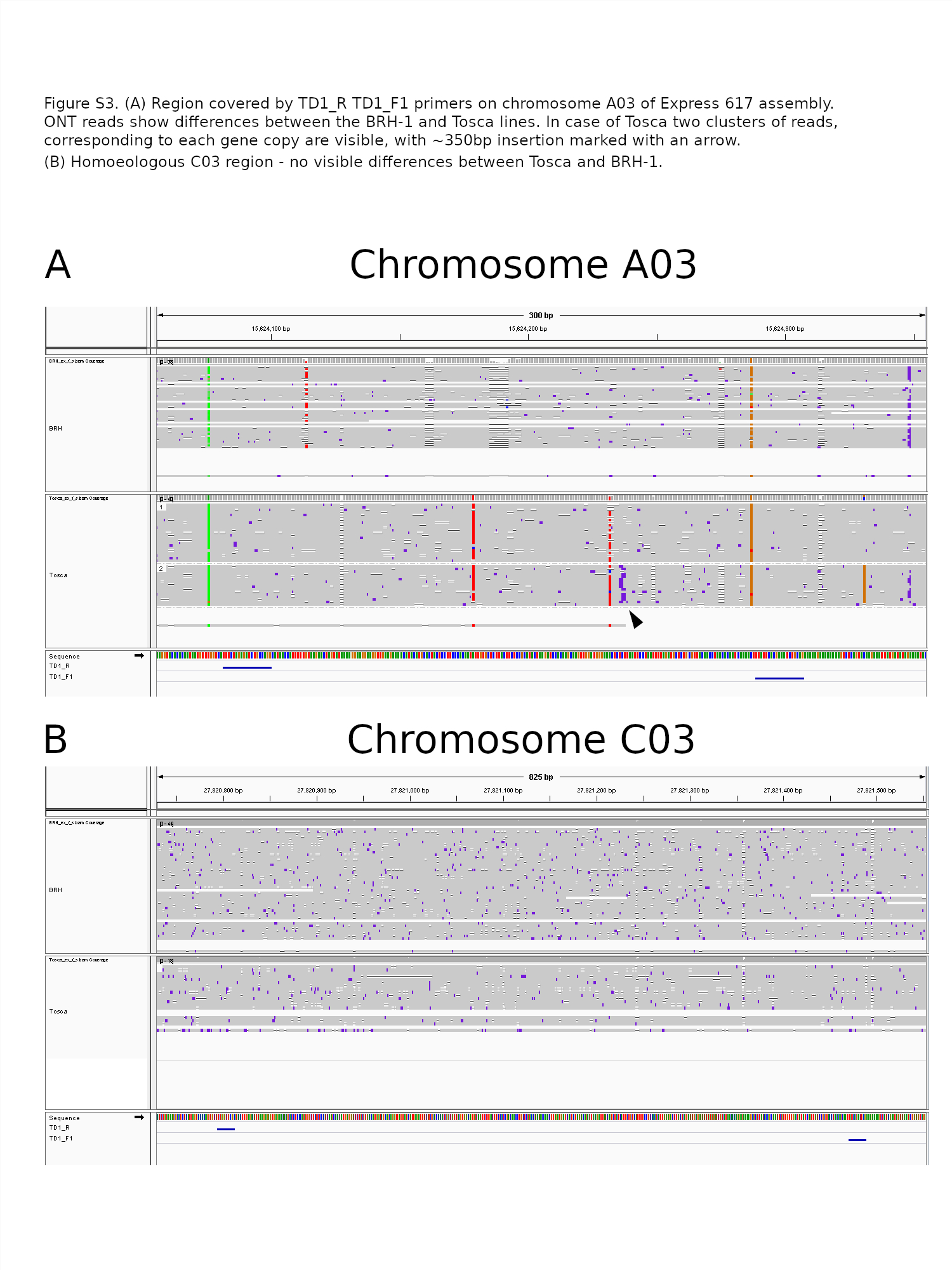

Supplement: Supplementary file 6 [file Image_3.JPEG]
